# Protocol for a randomised trial on the effect of group education on skin-protective behaviour versus treatment as usual among individuals with newly notified occupational hand eczema – the Prevention of Hand Eczema (PREVEX) Trial

**DOI:** 10.1186/1471-5945-13-16

**Published:** 2013-11-19

**Authors:** Maja Hvid Fisker, Tove Agner, Jane Lindschou, Jens Peter Bonde, Kristina Sophie Ibler, Christian Gluud, Per Winkel, Niels E Ebbehøj

**Affiliations:** 1 Department of Occupational and Environmental Medicine, Bispebjerg University Hospital, Copenhagen, Denmark; 2 Department of Dermatology, Bispebjerg University Hospital, Copenhagen, Denmark; 3 Copenhagen Trial Unit, Centre for Clinical Intervention Research, Rigshospitalet, Copenhagen University Hospital, Copenhagen, Denmark; 4 Department of Dermatology, Roskilde Hospital, Copenhagen University Hospital, Copenhagen, Denmark

**Keywords:** Occupational hand eczema, OHE, Occupational contact dermatitis (OCD), Prevention, Work related, Intervention

## Abstract

**Background:**

The incidence of occupational hand eczema is approximately 0.32 per 1,000 person years. The burden of the disease is high, as almost 60% has eczema-related sick leave during the first year after notification, and 15% are excluded from the workforce 12 years after disease onset. New treatments and prevention strategies are needed.

**Methods/Design:**

*Trial design:* The PREVEX trial is a randomised, parallel-group, superiority trial.

*Participants:* All individuals from the Capital Region of Denmark and Region Zealand with a suspected occupational skin disorder notified to the National Board of Industrial Injuries between June 2012 and December 2013 are invited to participate in the trial. Inclusion criteria are: self-reported hand eczema and informed consent. Exclusion criteria are: age <18 years or >65 years; permanent exclusion from the workforce; inability to understand the Danish language; any serious medical condition; and lack of written informed consent. We plan to randomise 742 participants. *Interventions:* The experimental intervention is an educational course in skin-protective behaviour and written information about skin care related to the participants' specific occupation. Also, a telephone hotline is available and a subgroup will be offered a work-place visit. The experimental and the control group have access to usual care and treatment. All participants are contacted every eighth week with questions regarding number of days with sick leave or other absence from work. 12 months after randomisation follow-up is completed. *Objective:* To assesses the effect of an educational course versus treatment as usual in participants with newly notified occupational hand eczema. *Randomisation:* Participants are centrally randomised according to a computer-generated allocation sequence with a varying block size concealed to investigators. *Blinding:* It is not possible to blind the participants and investigators, however, data obtained from registers, data entry, statistical analyses, and drawing of conclusions will be blinded. *Outcomes:* The three co-primary outcomes, assessed at 12 months, are: total number of self-reported days with sick leave; health-related quality of life; and subjective assessment of hand eczema severity. Explorative outcomes are: self-reported eczema-related sick leave, absence from work registered by the DREAM-register and by self-report, risk behaviour, knowledge of skin protection and performance management (self-efficacy; and self-evaluated ability to self-care).

**Discussion:**

The PREVEX trial will be the first individually randomised trial to investigate the benefits and harms of group-based education in patients with newly notified occupational hand eczema.

**Trial registration:**

ClinicalTrials.gov Identifier: NCT01899287

## Background

Occupational hand eczema is the most frequently recognised occupational disease in Denmark [1]. The incidence is approximately 0.32 per 1,000 persons in the total population [2], and the numbers are comparable in most of the industrialised world [3].

In 2011, the Danish National Board of Industrial Injuries recognised a total of 1,395 individuals with occupational skin diseases, and the large majority of these patients (> 90%) had occupational hand eczema.

Hand eczema affects mainly young people as 1/3 has onset of disease before the age of 20 years [2]. The lifetime prevalence is almost twice as high in women as it is in men [1]. This is mainly due to women’s higher exposure to wet work, occupationally as well as privately, which are well known risk factors [3]. Other aggravating factors are atopic dermatitis and contact allergy [1].

Hand eczema often takes a chronic course. A Swedish follow-up study reported active eczema 12 years after onset of disease in 72% of the sample of patients and found that 15% had been excluded from the work force [4]. In a Danish study, similar data were reported [5,6]. During a one-year period, 20% of patients with notified hand eczema had sick leave for more than five weeks due to skin disease, and 20% to 25% had loss of job (examined in the time span between notification and recognition). 57% had sick leave because of skin disease during the first year after notification. These data show that the burden of the disease is high in a personal as well as in a socio-economic context [7]. The number of persons with occupational hand eczema has remained unchanged despite public initiatives to reduce exposures to harmful allergens [8].

### Current treatment and prevention

In Denmark, there is no standard for secondary prevention of occupational hand eczema. However, operational guidelines for the diagnostic evaluation and treatment of hand eczema exist [9]. The guidelines recommend that advice should be given about personal protective equipment (e.g., gloves), and skin care with moisturisers.

In Germany, a course is offered to all health-care workers with occupational skin disease [10]. Persons with notified skin diseases are immediately after the notification invited to attend a two-day skin-protection course. The course is organised in cooperation with dermatologists, allergologists, occupational medicine specialists, hygiene specialists, and other staff members. The course includes a medical examination by a dermatologist, topics on medical history, atopic disposition, further diagnostics, therapy, skin protection, and an assessment of the patient’s ability to remain in her/his job. All findings by the physician are forwarded to the authorities, and they are the basis for the further treatment possibilities offered to the patient. This includes, in the most severe cases, initiation of a 3-weeks inpatient treatment programme. An observational study found that the course improved skin care and skin-protection behaviour, hand eczema, and quality of life one year after the course compared to before the course [11].

### Clinical data

A systematic review of randomised trials and controlled trials from 2010 assessed prevention programmes compared to no intervention or treatment as usual in participants with hand dermatitis [12]. The reviewers included seven trials. No meta-analyses were conducted, as the reviewers assessed the trials to be too heterogeneous with regard to populations, interventions, control groups, and outcome measures. Overall, the reviewers concluded that there was moderate evidence for the effect of preventive programmes on occurrence of hand dermatitis and adherence to preventive measures, and low evidence for improving clinical outcomes and self-reported outcomes. Furthermore, none of the included trials had ‘low risk of bias’ in all assessed domains, and the review was restricted to publications in English, with a high risk of publication bias. Also, the review did not distinguish between primary and secondary prevention trials.

We therefore conducted an updated search for randomised and controlled trials in hand eczema patients in December 2012 in the PubMed database. From this search, we identified two randomised trials with focus on secondary prevention by educational programmes in hand eczema patients. Both of these trials applied individual randomisation. One trial found that an individual intervention among health-care workers with self-reported hand eczema increases the participants’ use of preventive measures and improves hand eczema and quality of life compared with no intervention [13,14]. The other trial [15,16] found that patients with HE responded positively to integrated care carried out by a multidisciplinary team with respect to clinical severity, but the intervention did not significantly influence quality of life (QoL) or sick leave.

We identified nine other trials with focus on secondary prevention of hand eczema by educational programmes in various working groups. Overall, they have found a positive effect of *secondary* skin protection programmes on occupational hand eczema in patients from different professions such as health care workers [17-23], gut cleaners [24], and workers in the cheese-dairy industry [25]. However, since these studies did not focus on hand eczema patients, but on a mixture of healthy workers and workers with hand eczema, the results are not directly comparable. Also, most of these studies were cluster-randomised with a high risk of systematic errors (that is risk of overestimation of benefits and underestimation of harms) and random errors (that is play of chance), and two studies focused mostly on use of emollients [18,21]. In addition to the published trials we found one design article on a randomised trial [26]. This trial aims to investigate a guidance programme in self-management chronic hand eczema. The trial is ongoing and results are thus yet unknown.

Overall, randomised clinical trials and a systematic review of the randomised clinical trials indicate a positive effect of prevention programmes for patients with occupational hand eczema. However, we still lack information from large randomised clinical trials with a low risk of bias on the effect of a low-cost group-based education. The PREVEX trial will be the first individually randomised trial to investigate the benefits and harms of group-based education in patients with newly notified occupational hand eczema.

### Objectives

The objectives of the PREVEX trial are to investigate the benefits and harms of a complex intervention consisting of:

A. Group education on general skin-protective behaviour.

B. Job-specific counselling on work-related skin-protective behaviour, which might extend to a work-place visit.

C. Social guidance related to occupational hand eczema.

D. Telephone hotline for work- and patient-related problems, maintained by nurse or medical doctor.

## Methods

### Trial design

**Figure 1:**
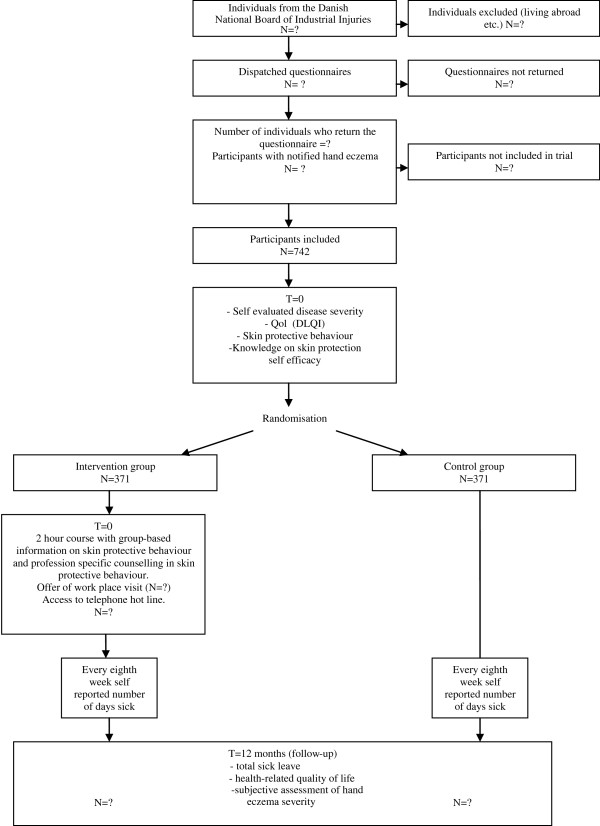
Trial flowchart.

The PREVEX trial is a randomised, parallel-group, superiority trial without blinded outcome assessment of the primary outcomes, but several of the secondary outcomes will be blinded (see below) (Figure 1).

### Setting and selection of participants

Participants are recruited from the National Board of Industrial Injuries files. These files include information on all notified industrial injuries in Denmark. All individuals from Region Zealand and the Capital Region of Denmark (except the island of Bornholm) with a suspected skin-related industrial injury notified between July 2012 and December 2013 will be invited to participate in the trial. The trial invitation will be sent along with the postal questionnaire, participant information sheet, an informed consent form, and a pre-paid return envelope. All individuals will be encouraged in the invitation to contact the investigators for further information or if they wish for an individual meeting with verbal information. Individuals who return the questionnaire will be eligible for the PREVEX trial, if they comply with the following inclusion and exclusion criteria.

### Inclusion criteria

• Living in Denmark.

• Self-reported hand eczema, i.e., individuals answering ‘yes’ in the questionnaire to the question ‘have you or have you had hand eczema?’

• The questionnaire is sufficiently filled in with respect to ‘severity of hand eczema’ and ‘profession’.

• Written informed consent.

### Exclusion criteria

• Age below 18 years or above 65 years.

• Permanently excluded from the workforce.

• Inability to understand the Danish language sufficiently to benefit from the course.

• Any serious medical condition which, in the opinion of the investigator, may interfere with the evaluation of the results.

• Lack of written informed consent.

### Participant withdrawal and discontinuation of participants

The participants are free to withdraw their informed consent from the trial at any time without effecting future treatment or their case processing in the National Board of Industrial Injuries. If a participant wishes to withdraw his or hers consent, they will be contacted and asked to specify which aspects of the trial they wish to withdraw from: participation in the experimental intervention, participation in follow-up interviews and answering the questionnaires, use of follow-up data collected from national databases, or complete withdrawal from trial, i.e., use of already collected data the analyses.

All participants who enter the trial will be accounted for in the publication of the results. Participants who fail in attending a course on a planned date will be contacted and offered a new course. Participants who cannot be reached during the follow-up period will be contacted and encouraged to resume contact. Participants who do not return the follow-up questionnaire will be contacted and a letter will be sent containing a questionnaire on reasons for drop out.

### Randomisation

When the participants have returned the questionnaire and they fulfil all inclusion criteria and none of the exclusion criteria, they are randomised. The investigator contacts the Copenhagen Trial Unit (CTU) by telephone, and the CTU staff will perform the allocation according to data entered in a computer system. Participants will be randomised individually 1:1 to the experimental group versus the control group centrally according to a computer-generated allocation sequence with a varying block size concealed to investigators. Randomisation will be stratified according to age (up to 39 years, or older than 40 years); self-reported hand eczema severity (‘none and light’ or ‘moderate, severe, and very severe’); and profession (‘healthcare’, ‘kitchen or cleaning staff’, ‘hairdresser or construction worker’, or ‘all other professions’). After the allocation, the investigators will inform the participants about the allocation by telephone.

### Blinding

It is not possible to blind the participants or investigators with respect to treatment allocation. However, data entry from the questionnaires, outcome assessment regarding absence from the work force (the eight week follow-up telephone calls, data from the DREAM register, and data from other national registers), statistical analyses, and conclusions will all be blinded.

### Trial duration

The inclusion period is estimated to be 18 months. The first participant was included in 13 July 2012, and we expect inclusion of the last participant in December 2013. Self-reported data on absence from work will be continuously collected (every eighth week). Follow-up for the individual participants will be 12 months after inclusion and is expected to be completed in December 2014.

### Experimental group

The experimental intervention consists of four components, A to D, as well as usual care, E:

A. *Group education on general skin-protective behaviour*

Participants in the experimental group will participate in a two-hour course. One or two courses per week will be available. A secretary or nurse will arrange booking with the participants allocated to the experimental group. A maximum of 12 participants per course is expected. The courses will be conducted by two investigators. Participants can choose between an evening and a daytime course.

The group education will comprise basic knowledge about the skin, the development of eczema, and recommendations for skin protection and care. The recommended skin protection programme has been summarized into ten recommendations in Denmark (Table 1). These ten recommendations will be the basis of the education [27].

There will be practical demonstrations in hand washing and use of disinfectants, use of moisturiser, and use of different types of gloves (cotton, rubber, nitrile, vinyl gloves, and gloves with and without fingers).

B. *Job-specific counselling on work-related skin-protective behaviour, which might extend to a work-place visit*

The job-specific counselling will comprise specific recommendations for skin care in different occupations. For ten occupations with some of the highest numbers of recognised occupational hand eczema patients in 2010 [28], the information will be standardised and written information will be available (Table 2). In case of other occupations, or with specific problems, a work visit will be offered and arranged. A doctor or a nurse will undertake the visit, and counselling will be based on this.

C. *Social guidance related to occupational hand eczema*

The social guidance related to the occupational hand eczema will include information on rules and rights during an occupational injury. This will also include information about the options for help from workers compensation or social welfare in case of job-loss or change.

D. *Telephone hotline*

An occupational nurse will provide assistance via telephone about questions and social problems. The telephone hotline will mainly include information already given at the course, but may be more specifically given to the particular individual. The purpose of the hotline is to repeat information from the course, which may have been forgotten or misunderstood, and to guide the participant in case of questions related to the social situation related to the occupational skin disease. It is the intention that the hotline shall generally function as a ‘helping hand’.

E. *Treatment and care as usual*

The experimental group will have access to usual treatment and care (see below).

**Table 1:** **The ten recommendations regarding how to avoid hand eczema**[27]

1.	Use gloves when beginning wet work tasks.
2.	Gloves must be used as long time as necessary but as short as possible.
3.	Protective gloves should be intact and clean and dry inside.
4.	When protective gloves are used for more than 10 minutes, cotton gloves should be worn underneath.
5.	Wash hands in lukewarm water and dry them well.
6.	Alcohol-based disinfections should be used instead of soap when the hands are not visibly dirty.
7.	Do not wear rings at work.
8.	Use a moisturiser with a high fat content and no perfume.
9.	Moisturisers should be applied all over the hands, including the webs, finger tips and dorsal aspects.
10.	Take care also when doing domestic work. Use protective gloves for dish washing and insulating gloves in the winter.

**Table 2:** **Ten occupations where job-specific recommendations for skin care is available**[28]

**Occupation**	**Number of recognised occupational hand eczema patients in 2010**
Health care workers, care workers not hospital	456
Kitchen workers/chef, baker, butcher	214
Cleaners	118
Craftsmen	105
Hairdresser	101
Factory worker	88
Mechanics, machine operator, metal worker	113
Sales assistant	41
Dentist assistant	39
Gardener, agricultural worker	37

### Control group

The control group will not have access to the components A to D encompassing group education, the profession specific information, the social guidance, and the telephone hotline, but they have access to usual treatment and care with their general practitioner and dermatologist (component E).

### Usual treatment and care available to both intervention groups

After notification to the National Board of Industrial Injuries, a medical examination including allergy testing of the patient will take place. This will be either as a specialist’s certificate performed by a specialist in dermatology, or an examination in a dermatological department in a hospital. The decision to recognise or reject the patient as having an occupational disease will be based on data from this investigation.

The participants in both intervention groups have access to the usual care and treatment with the general practitioner and dermatologist. Such care will be registered via central registers.

### Concomitant medication and treatment

All medication and other treatment are permitted during the trial. We will register use of topical corticosteroids and visits with a dermatologist for all participants in the follow-up period.

### Data collection

#### Inclusion

Participants fill in a questionnaire regarding basic demographic factors, self-reported level of eczema severity using a photographic guide [29], health-related quality of life (the Dermatology Life Quality Index) [30], level of knowledge concerning prevention of hand eczema, occupational exposure and exposure during leisure time, strength of coping, and performance management. The questionnaires are returned by mail. Data will be entered into Statistical Analysis Software (SAS). This will be done by scanning and where that is not possibly, manually, by external assistants.

#### Participation in the experimental intervention

Participation in the course will be registered in paper record forms by the investigators and later manually registered in SAS.

#### Follow-up period, from inclusion to 12 months after inclusion

Data on absence from workforce will be collected every eight weeks, by telephone call, e-mail, text message or any other chosen form of media by a blinded investigator, for one year in the intervention group and in the control group. The data will be registered directly in paper record forms by a nurse. Absence will be categorised by participants as: total sick leave; eczema-related sick leave; absence from work for other reasons (child care during illness, etc.); unemployment; and other reasons. Later these data will be entered into SAS by the external assistants.

#### End of follow-up, 12 months after inclusion

Participants fill in a follow-up questionnaire regarding self-reported level of eczema severity using a photographic guide [29], health-related quality of life (the Dermatology Life Quality Index) [30], level of knowledge concerning prevention of hand eczema, occupational exposure and performance management. The follow-up questionnaire will be distributed by mail to all participants and when returned, entered into SAS by scanning and where that is not possibly, manually, by external assistants. Data on absence from the work force for a period longer than 28 days will be collected from the DREAM register [31]. Further, data regarding potential effect modifiers, i.e., the participants’ use of topical corticosteroids will be collected from The Danish National Database of Reimbursed Prescriptions [32], and visits with a dermatologist will be retrieved from The National Health Insurance Service Registry [33]. Data from both national registers will be collected using the unique Danish Civil Registration Number.

### Data management

Data is handled and recorded in paper record forms and kept in records marked with investigator number, patient identification number, and name of region. The data on absence from work are handled and recorded in participant record forms and kept in records marked with investigator number, patient identification number, name of region, and time of assessment. Paper questionnaires collected at follow up (12 months) is kept in other records marked with investigator number, name of region, and time of assessment. After follow up all the record forms from each participant will be collected in individual files and kept in records. Any change in the files or participant record forms will be documented with date and signature of the investigator.

Data from the records will be registered electronically in SAS for statistical analyses. This will be done partly by scanning and partly manually by external assistants. Records will be archived for at least five years after end of trial.

### Quality control and quality assurance

To ensure the trial is conducted and reported in compliance with this protocol, the data are monitored internally. The investigators monitor the data and check for systematic errors. All data are registered in paper record forms and kept at an investigator site file available only for the investigators. Around 5% of the data is monitored to assess consistency between the paper record forms, electronically registered data, and signed consent forms. Data are handled with confidentiality.

### Primary outcomes

The PREVEX trial has three co-primary outcomes:

• **Total sick leave**; measured as self-reported total number of days with sick leave during the trial period.

• **Health-related quality of life;** measured as points scored in the Dermatology Life Quality Index (DLQI) at 12 months after inclusion [30].

• **Subjective assessment of hand eczema severity**; measured by use of a photographic guide at 12 months after inclusion [29].

### Explorative outcomes

The following outcomes will be assessed as explorative, as there is insufficient information to conduct power calculations.

• **Eczema-related sick leave;** measured as self-reported total number of days with eczema-related sick leave during the trial period.

• **Absence registered by the DREAM-register**; only absence for more than 28 days from workplace is registered here. This will be done at 12 months after randomisation. We will measure absence from work because of sick leave for more than 28 days, yes or no.

• **Absence, self-reported** total number of days with absence during the trial period.

• **Behaviour** measured as number of points achieved in a questionnaire concerning both occupational and private risk behaviour at 12 months after randomisation.

• **Knowledge of skin protection** measured as numbers of points achieved in a multiple choice questionnaire at 12 months after randomisation.

• **Performance management** at 12 months after randomisation of the participant measured by the number of points achieved in:

O Self-efficacy [34]; and

O Self-evaluated ability to self-care.

### Risks and benefits

We do not expect any risks for the participants, except the risks following transportation to and from the educational site. The intervention is of educational character, no drugs or medical devices will be used. During the course there will be a nurse and/or medical doctor present. In case of adverse advents, the investigators will act according to their professions’ current ethical and professional standards.

If the intervention proves effective, benefits such as lower severity of occupational hand eczema, less sick leave, and diminished risk of exclusion from the work force are expected in the experimental group. A general improvement in health-related quality of life is also expected.

### Ethical considerations

The intervention is of educational character. The intention is to improve knowledge, behaviour, and consequently disease severity and absence from work, by an educational programme. No drugs or risk-full testing are used in the trial.

Medical treatment of the patients hand eczema will not be a part of this trial, and there will be no difference in access to the medical treatment in the experimental and control group.

Furthermore, as there is no existing evidence on the effect of more expensive interventions in this patient group, it is considered good ethical conduct to perform the described trial with a control group who are offered ‘treatment as usual’.

The main results of the trial will be presented to the included participants in a newsletter, where there will be references to any data published in medical journals.

### Trial conduct

The PREVEX trial will be conducted in compliance with the trial protocol and the Helsinki Declaration in its latest form. The protocol has been submitted for review to the Regional Ethics Committee for the Capital Region (journal number: H-1-2012-053), who replied that the PREVEX trial is not a ‘biomedical trial’, as no medicinal interventions take place, and accordingly a formal review by the Ethics Committee is not necessary. The trial has been approved be the Danish Data Protection Agency (journal number BBH-2011-33), and it has been registered on www.clinicaltrial.gov (identifier: NCT01899287).

### Sample size estimation

Calculations were made using ‘PS: Power and Sample Size Calculation’ version 3.0.14 [35]. A sample size was calculated for each of the three co-primary outcomes and the largest of the three sample sizes was chosen as the target sample size. To adjust for multiple testing, we divided the risk of type I error, 5%, by three, and we thus used a risk type I error of 1.67% and a risk of type II error of 20% (80% power). This calculation is based on the assumption that Holm’s procedure [36] will be used to adjust alpha (5%) to ensure that the family wise type I error will not exceed 5%. Holm’s procedure works as follows: let p(1), p(2) and p(3) be the observed P values where p(1) < p(2) < p(3) and H(1), H(2) and H(3) are the corresponding hypotheses. The hypotheses are tested in that order. At the J’th step H(J) is rejected if P(J) < 0.05/(3 – J +1). Otherwise H(J) and all other untested hypotheses are accepted and the procedure stops. Since we cannot know in advance which of the three outcomes will be associated with the smallest P value, the largest of the three sample sizes is chosen to secure that the power will be at least 80%.

For the continuous variable ‘total sick days’, we used unpublished data from the Working Environment Cohort (Arbejdsmiljøkohorten) from the National Research Centre for the Working Environment (http://www.arbejdsmiljoforskning.dk) to estimate the sample size. In this cohort of 9,786 healthy individuals, the mean number of sick days in year 2010 was 6.74 (95% confidence interval (CI) 6.32 to 7.15). We expect a higher degree of sick days in the PREVEX population, and we estimate that the mean number of total sick days in the PREVEX trial will be higher, probably about 20 sick days per year.

From the data above, we calculated the standard deviation (SD) using this formula:

$$\begin{array}{c} \mathrm{SD}=\sqrt{N}\times \frac{95\%\mathrm{CI}\;\mathrm{upper}\;\mathrm{limit}-95\%\mathrm{CI}\;\mathrm{lower}\;\mathrm{limit}}{3.92}\\ ⇓\\ \mathrm{SD}=\sqrt{9,786}\times \frac{7.15-6.32}{3.92}\\ ⇕\\ \mathrm{SD}=21.0 \end{array}$$

To detect a difference in total sick days of 5 days (25% risk reduction), we need 742 participants (371 participants in each intervention group) (Table 3).

**Table 3:** Sample size estimations

**Outcome**	**Minimum relevant difference**	**Risk of type I error**	**Risk of type II error**	**Total number of participants needed**
Total sick days	5 days (SD* 21 days)	1.67%	20%	742
DLQI^†^	1.2 points (SD 4.8 points)	1.67%	20%^‡^	672
Subjective assessment of hand eczema severity	20% risk reduction from 60% event rate in the control group	1.67%	20%^§^	720

*Standard deviation of the mean.

^†^Dermatology Life Quality Index.

^‡^Will be reduced to 15.7% when 742 participants are included.

^§^Will be reduced to 18.6% when 742 participants are included.

For the DLQI, we used a standard deviation of 4.8 points [37]. In order to detect a difference between the two intervention group of 1.2 points (corresponding to 25% of the standard deviation), we will need to include 672 participants (336 participants in each intervention group). By inclusion of 742 participants, the risk of type II error will be reduced to 15.7% (power of 84.3%).

For the subjective assessment of hand eczema severity, prior data indicate that the proportion of participants with moderate to severe symptoms is 60% [38]. If the proportion of participants with moderate to severe symptoms can be reduced to 48% (relative risk reduction of 20%) in the experimental group, we need to include 720 participants (360 participants in each intervention group). By inclusion of 742 participants, the risk of type II error will be reduced to 18.6% (power of 81.4%).

In total, we will include 742 participants in the PREVEX trial, which was the maximum number of participants needed according to the three calculations.

### Expected participant recruitment

A former study using a questionnaire on patients with occupational skin disease conducted in 2002 achieved a high response rate of 82% [6]. According to statistics from the National Board of Industrial Injuries, approximately 1,000 individuals reported a skin-related industrial injury in 2010 in Region Zealand and in the Capital Region of Denmark. Therefore, we conservatively assume that we will be able to complete recruitment of the 742 participants within 18 months.

### Plan for statistical analyses

#### Analysis of the three primary outcomes

##### Types of analyses

Depending on the specific type of outcome measure, one of three types of regression analyses will be applied.

• *Type 1* includes a rate (count of events over period of observation (12 months according to plan). Using the countreg procedure (SAS 9.3), the Poisson model, and the negative binomial model, the rate will be compared by testing for overdispersion and comparing the average predicted count probabilities and the observed proportions. The best model will be used to analyse data.

• *Type 2* includes a continuous outcome measure. The general linear univariate model will be used.

• *Type 3* The ordinal outcomes will be analysed using the proportional odds model where the intervention group indicator is included as a co-variate provided the P value of the score test of the proportional odds assumption is ≥ 0.05.

All of the above analyses will include the three protocol specified stratification variables (age groups, self-reported hand eczema severity, and profession) as covariates in addition to the intervention indicator and baseline value if available. If the assumptions of an analysis are seriously violated, a non-parametric test will be used.

##### Missing values

If the percent participants with missing values exceeds 5% for any of the three outcomes or Little’s test is significant (p < 0.05), multiple imputations (MI) will be used (SPSS version 17 or later).

##### Sensitivity analyses

For each of the three outcomes, two types of imputations will be conducted. 1) Missing values in one group will be imputed by the maximum value observed in the material and missing values in the other group will be imputed by the minimum value observed, and 2) vice versa. For each type of imputation the two groups will be compared to obtain the spectrum of potential bias.

##### Multiplicity

The three P values corresponding to the null hypotheses of the success criterion will be adjusted using Hommel’s procedure [39]. Hommel’s procedure could not be assumed in the sample size calculation since the level of significance to be used cannot be known in advance and therefore the adjustment of alpha could not be specified. However, Hommel’s procedure is uniformly more powerful than Holm’s procedure [36]. So the reduction of the level of significance will be less than or equal to that used were the Holm procedure to be used.

#### Exploratory analyses

The exploratory analyses include regression analyses of continuous and rate data observed 12 months following randomisation. They are all similar to the above described analyses. In addition, every 8 weeks during the first 40 weeks and then at week 52 following the randomisation the rate of sick leave is calculated. The six rates are referred to as rate-I (I = 1 to 6) in the following where we refer to the above six periods with rate-1 being the rate observed during the first 8 weeks, rate-2 the rate observed during week 9 to 16 following randomisation, etc. The rate is defined as the number of days with sick leave divided by the number of days of observation. Using the generalised linear mixed model with repeated measures we want to study rate as a function of time T, the intervention indicator, and the covariates where T varies from 1 corresponding to period 1 (first 8 weeks) to 6 (last 12 weeks) and rate = rate-I for T = I.

### Trial organisation

The trial will take place at Bispebjerg Hospital in the Capital region of Denmark and at local hospitals where suitable in Region Zealand. The investigators are responsible for the protocol, conducting of the trial, and all other aspects involved.

### Finance and insurance

The trial is financed by job-specific who covers all expenses related to the trial (grant number: 20.2010-09). The participants in the trial are covered by the Patients Insurance Association under the existing rules as the trial is performed under the authority of Bispebjerg Hospital.

### Publication plan

The trial results will be published in international dermatological, medical and occupational scientific journals and presented in national and international dermatology and occupational medicine conferences. The investigators will follow the rules and guidelines of the International Committee for Medical Journal Editors (ICMJE) for authorship [40].

## Discussion

The prevalence of occupational hand eczema is high with large consequences at the personal and societal level. The ultimate aim of the PREVEX trial is to contribute to the development of new strategies for secondary prevention in patients with occupational hand eczema.

The timing in this trial is unique. Notification of an occupational injury often brings the individual in an insecure situation concerning the affiliation with the workforce. The intervention is delivered immediately after notification, which gives the best conditions to minimise these elements. The intervention in PREVEX is general as well as simplistic. This will heighten the possibility to determine the actual effectors, if any, in the intervention. Furthermore, the intervention is low-cost and thus more implementable in society.

The PREVEX trial will be a pragmatic trial, as the trial population will be heterogeneous regarding the severity of hand eczema, participants’ occupation, and previous treatment ranging from none to care in a dermatological setting. Accordingly, findings of the trial should have generalisability.

The trial has been designed in order to minimise the risks of systematic errors [41-43] and the risks of random errors [43]. Systematic errors have been sought reduced by central randomisation stratified for prognostic factors [41-43]. Blinding is used whenever possible and data will be analysed according to the intention-to-treat principle. We are aware that some outcomes are at risk of being assessed with some bias, as they are not possible to blind [41-43].

The risk of random error has been reduced by choosing the largest of three possible sample size estimations. Any significant differences regarding the exploratory outcomes shall be interpreted with caution as they may be due to random errors.

## Competing interests

The authors have no financial or academic competing interests in relation to the trial.

## Authors’ contributions

MF: Responsible for inclusion of patients, participated in the courses given for the patients, and drafted the manuscript. TA: Conceived of the trial, participated in design of the intervention (the courses given for the patients) and helped to draft the manuscript. JL: Drafted the manuscript, participated in design and coordination of the trial, practically responsible for the randomisation and sample size estimations. JPB: Participated in the design of the trial and design of the statistical analyses. KSI: Participated in the design of the trial and coordination of the trial. CG: Participated in the design of the trial, design of the randomisation procedure, and writing of the statistical analysis plan. PW: participated in the design of the trial and in writing of the statistical analysis plan. NEE: Conceived of the trial, participated in design and coordination and helped to draft the manuscript. All authors read and approved the final manuscript.

## Pre-publication history

The pre-publication history for this paper can be accessed here:

http://www.biomedcentral.com/1471-5945/13/16/prepub
